# Explaining the heterogeneity of functional connectivity findings in multiple sclerosis: An empirically informed modeling study

**DOI:** 10.1002/hbm.24020

**Published:** 2018-02-21

**Authors:** Prejaas Tewarie, Martijn D. Steenwijk, Matthew J. Brookes, Bernard M. J. Uitdehaag, Jeroen J. G. Geurts, Cornelis J. Stam, Menno M. Schoonheim

**Affiliations:** ^1^ Sir Peter Mansfield Imaging Centre, School of Physics and Astronomy University of Nottingham Nottingham United Kingdom; ^2^ Department of Neurology Amsterdam Neuroscience, VUmc MS Center Amsterdam, VU University Medical Center Amsterdam the Netherlands; ^3^ Department of Anatomy and Neurosciences Amsterdam Neuroscience, VUmc MS Center Amsterdam, VU University Medical Center Amsterdam the Netherlands; ^4^ Department of Clinical Neurophysiology and MEG center Amsterdam Neuroscience, VU University Medical Center Amsterdam the Netherlands

**Keywords:** biophysical modeling, functional connectivity, functional networks, grey matter damage, multiple sclerosis, white matter damage

## Abstract

To understand the heterogeneity of functional connectivity results reported in the literature, we analyzed the separate effects of grey and white matter damage on functional connectivity and networks in multiple sclerosis. For this, we employed a biophysical thalamo‐cortical model consisting of interconnected cortical and thalamic neuronal populations, informed and amended by empirical diffusion MRI tractography data, to simulate functional data that mimic neurophysiological signals. Grey matter degeneration was simulated by decreasing within population connections and white matter degeneration by lowering between population connections, based on lesion predilection sites in multiple sclerosis. For all simulations, functional connectivity and functional network organization are quantified by phase synchronization and network integration, respectively. Modeling results showed that both cortical and thalamic grey matter damage induced a global increase in functional connectivity, whereas white matter damage induced an initially increased connectivity followed by a global decrease. Both white and especially grey matter damage, however, induced a decrease in network integration. These empirically informed simulations show that specific topology and timing of structural damage are nontrivial aspects in explaining functional abnormalities in MS. Insufficient attention to these aspects likely explains contradictory findings in multiple sclerosis functional imaging studies so far.

## 1 INTRODUCTION

Cognitive deterioration is common in multiple sclerosis (MS) (Chiaravalloti & DeLuca 2008), but poorly understood (Benedict & Zivadinov 2011; Rocca et al., 2015a). Previous functional imaging studies in MS hypothesized that a process called “functional reorganization” compensates for accumulating structural damage before the functional network collapses, leading to inadvertent cognitive decline (Schoonheim, Meijer, & Geurts, 2015b). Interestingly, however, studies investigating connectivity changes demonstrate contradictory results: both increases and decreases in connectivity are reported to correlate with cognitive decline (Schoonheim et al., 2015b). Therefore, it is now crucial to elucidate how both increased and decreased connectivity can arise in the same set of patients in different disease stages, and how both can relate to worse cognitive outcomes at the same time. We postulate that the reason for such contradictory results can only be elucidated in a completely controlled system.

Discrepancy between results on connectivity in MS may be driven by several factors (Stam 2014). Heterogeneity is one factor that could play a major role, both heterogeneity in demographic factors and disease‐specific factors. For example, studies differ with respect to proportion of disease types in the population of interest, disease duration, female versus male ratios, age, physical status, but also in terms of MRI characteristics, for example, the amount of diffuse white matter damage or grey matter atrophy. Other more technical factors may also have contributed to divergent results on connectivity and activity in MS, for instance, suboptimal processing of fMRI data and suboptimal statistics using well‐known functional imaging toolboxes (Eklund, Nichols, & Knutsson, 2016).

However, another more fundamental hurdle is a lack of understanding how functional connectivity changes in MS relate to structural abnormalities, such as thalamic or cortical atrophy, assumed to reflect loss of neuronal connections and neurons (Popescu et al., 2015). We hypothesize that understanding the relationship between structure and function in MS could elucidate how discrepant connectivity results may emerge in a disease with ubiquitous heterogeneity. Disentangling the separate effects of white matter (WM) and grey matter (GM) pathology on connectivity and networks from empirical data is currently challenging due to several factors such as lack of longitudinal data, limited sample sizes, heterogeneity in patient groups, use of different MRI scanners with different field strength and hardware, and differences in imaging pipelines. Biophysical models, mimicking electrophysiological signals, have been used successfully to understand pathophysiological processes in several other neurological diseases (Breakspear et al., 2006; Modolo et al., 2010; Moran et al., 2011; van Dellen et al., 2013). These models allow for realistic longitudinal data simulations that may help to understand the effects of demyelinating and neurodegenerative MS pathology on functional connectivity and functional network organization. In other words, at this stage, these models are both an adequate and a necessary alternative for longitudinal empirical data and allow analyzing the influence of structural damage (in its various forms) on the functional network in a completely controlled and systematic way.

In this study, we employ a biophysical model, informed by empirical data, to investigate the separate effects of white matter, cortical, and thalamic degeneration on functional connectivity and network organization in MS. We hypothesize that the stage and amount of grey and white matter MS pathology can elucidate the contradictory results that were previously explained as “functional reorganization.”

## 2 METHODS

### 2.1 Cortico‐thalamic mean field model

**Figure 1:**
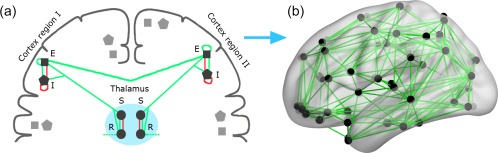
Overview of the cortico‐thalamic mean field model. Panel (a) shows two units (region I and II), consisting of a cortical excitatory (E), cortical inhibitory (I), thalamic relay (S), and thalamic reticular (R) populations. These populations are linked by within‐unit and between‐unit connections (e.g., the green line that crosses the midline between the two hemispheres). Connections can either be excitatory (green) or inhibitory (red). The connection between the thalamus and the cortex is reciprocal. The cortical parts of the units are sampled over the cortex and only the excitatory populations are connected between units using an empirically informed anatomical network (panel b). Note that these connections are green and thus excitatory [Color figure can be viewed at http://wileyonlinelibrary.com]

Given the strong clinical relevance of cortico‐thalamic involvement for cognition in MS (Houtchens et al., 2007; Schoonheim et al., 2015a), we employ a well‐known cortico‐thalamic mean field model in this study (Abeysuriya, Rennie, & Robinson, 2014; Robinson, Loxley, O'connor, & Rennie, 2001; Robinson, Rennie, Rowe, & O'Connor, 2004). This model is informed by empirical data and optimized to produce realistic power spectra mimicking electroencephalogram/magnetoencephalography recordings. It is also one of the few large‐scale biophysical models that include the thalamus. In short, the model consists of units, where each unit comprises two cortical (excitatory and inhibitory) and two thalamic (relay nuclei and a reticular nucleus) populations (Figure 1a). We denote these groups as a∈{e,i,r,s}, where e,i,r,s denote excitatory, inhibitory, reticular, and relay, respectively. For each group, a, and region j, the mean membrane potential is denoted by $V_{a,j}$ and the mean firing rate by $Q_{a,j}$, which were interrelated by a sigmoid function:

(1) $$Q_{a,j}=\frac{Q_{max}}{1+exp(-(V_{a,j}-\theta )/\sigma ))}$$

Here, $Q_{max}$ refers to the maximum firing rate in Hz, θ is the mean firing threshold in mV, and σ is the standard deviation of this threshold. The mean membrane potential $V_{a,j}$ itself fluctuates under the influence of incoming firing input from other groups within the same population ${(Q}_{a,j}$, a∈{e,i,r,s}) and from other excitatory regions belonging to other populations, $\phi_{e,n}$, outside its own region. The mean membrane potential $V_{a,j}$ can be considered as a low‐pass filter. For every group, its dynamics can be described by

(2a) $$D_{a}V_{e,j}\left(t\right)=v_{ee}\phi_{e,j}\left(t\right)+v_{ei}Q_{i,j}\left(t\right)+v_{es}Q_{s,j}\left(t-\tau_{ct}\right)+\epsilon \frac{1}{N}\sum_{n=1,\;n\neq j}^{N}A_{jn}\phi_{e,n}(t-\tau_{jn})$$

(2b) $$D_{a}V_{i,j}\left(t\right)=v_{ie}\phi_{e,j}\left(t\right)+v_{ii}Q_{i,j}\left(t\right)+v_{es}Q_{s,j}\left(t-\tau_{ct}\right)$$

(2c) $$D_{a}V_{s,j}\left(t\right)=v_{se}\phi_{e,j}\left(t-\tau_{ct}\right)+v_{sr}Q_{r,j}\left(t\right)+v_{n}\phi_{noise,j}(t)$$

(2d) $$D_{a}V_{r,j}\left(t\right)=v_{re}\phi_{e,j}\left(t-\tau_{ct}\right)+v_{rs}Q_{s,j}\left(t\right)$$

(2e) $$D_{a}=\frac{1}{\alpha \beta }\frac{d^{2}}{dt^{2}}+\left(\frac{1}{\alpha }+\frac{1}{\beta }\right)\frac{d}{dt}+1.$$

Here α and β are constants, which are independent of time and correspond to the synaptic rise and decay rates in s^−1^; $v_{aa}$ denotes the synaptic densities between the population types and ϵ corresponds to the global structural coupling strength between populations. The last term in Equation (2c) corresponds to noise, which is defined as $\phi_{noise,j}(t)=\sigma_{n}\alpha \beta \chi \left(t\right)$, where $\chi \left(t\right)$ denotes a unit variance Gaussian white‐noise process, and $\sigma_{n}$ is the strength of this process. A number of cortical populations is sampled over the cortex and connected to each other through empirically informed white matter connections (Figure 1b) (Gong et al., 2009), which is of the same size as previously described empirical networks in MS (Tewarie et al., 2014a, 2013, 2014b; Tewarie, van Dellen, Hillebrand, & Stam, 2015). Therefore, external firing input from other populations is mediated by the presence of an structural connection $A_{jn}$, where A denotes a 78 × 78 adjacency matrix of a literature based structural network (Gong et al., 2009). Input from the thalamus to the cortex and vice versa is delayed by $\tau_{ct}$, and cortico‐cortical excitatory input between regions is also mediated with a delay $\tau_{jn}$, depending on the Euclidian distance in the AAL atlas between region *j* and *n*. As incoming excitatory firing input from other populations $\phi_{e,n}$ is propagated over long range white matter tracts, its initial activity $Q_{e,n}$ is damped by the following expression

(3) $$\left(\frac{1}{\gamma^{2}}\frac{d^{2}}{dt^{2}}+\frac{2}{\gamma }\frac{d}{dt}+1\right)\phi_{e,n}\left(t\right)=Q_{e,n}(t)$$

The constant γ refers to the cortical damping rate. Equation (3) usually has an extra term on the left hand side, which contains the differential Laplace operator $\nabla^{2}$. Similar to Robinson, Rennie, and Rowe (2002), we ignore the spatial dynamics by setting $\nabla^{2}=0$ and therefore all observables in the model become independent of position in space. Note that there is still spatial dependence based on the incoming connections over the white matter tracts in the network (Equation (2a)). Values for all constants can be found in Supporting Information, Table S1 and are the same parameters as used to simulate resting‐state activity in previous studies (Hindriks & van Putten 2012, 2013; Robinson et al., 2002), with the only exception that we increased $v_{ei}$ to account for the extra excitatory input from the network. Simulations based on the cortico‐thalamic mean‐field model were performed using the Euler–Maruyama method with an integration time step of 1 × 10^−4^. The first 2 s (20,000 samples) of the simulated data were discarded to exclude any non‐oscillatory data. Only the time series of the 78 excitatory cortical populations $\phi_{e,n}\left(t\right)$ were used as the model output to mimic MEG signals (Robinson et al., 2002), as it is believed that measurable and empirical neurophysiological signals outside the head mainly originate from excitatory pyramidal neurons (Hämäläinen, Hari, Ilmoniemi, Knuutila, & Lounasmaa, 1993). Note that the thalamic populations are required to generate realistic alpha band oscillations in a thalamo‐cortical loop (Hindriks & van Putten, 2013); however, they are not used as model output to mimic MEG signals since they are usually difficult to detect using MEG. Thus, signals from excitatory cortical populations were used and filtered in the alpha band (8–13 Hz), which is the dominant frequency band of the model, but also the frequency band which most often shows cognitively relevant altered connectivity and disrupted network organization in MS (Cover et al., 2006; Schoonheim et al., 2013; Tewarie et al., 2014a, 2013, 2014b). Analysis of the signals from the excitatory populations enables us to study a realistic system of coupled brain regions in terms of their activities and functional connectivity between them.

### 2.2 Outcome measures

We define four outcome measures: mean activity, mean functional connectivity, network diameter, and leaf fraction. (a) Mean *activity* is defined as the average magnitude of the alpha band activity across all excitatory cortical populations, where strength of the activity corresponds to the spectral power of the alpha band. (b) Mean functional *connectivity* is defined as phase synchronization measured by the phase locking value (PLV) (Lachaux, Rodriguez, Martinerie, & Varela, 1999).The PLV assumes that there is high connectivity when the phase difference of two signals is stable over a certain amount of time, and was used to estimate alpha band connectivity between all possible region pairs to obtain a weighted functional connectivity matrix. We subsequently average across regions to obtain a single average PLV value for each simulation.

In addition, we examined network organization corresponding to the functional connectivity matrix obtained in the previous paragraph. The difference between *functional connectivity* and *functional network organization* is that functional connectivity refers to the *strength* of connections between brain regions, whereas functional network organization (topology) corresponds to the *pattern of connections* between regions in the brain. Specifically, we assess network organization by computing two network topology measures based on the minimum spanning tree (MST) (Stam et al., 2014; Tewarie et al., 2015b): diameter and leaf fraction. (c) *Diameter* is defined as the longest shortest path within the MST and (d) *leaf fraction* refers to the fraction of regions in the MST with only one connection. Here, diameter captures information about network integration (i.e., the smaller the diameter, the more the network is integrated), while leaf fraction captures information about segregation. The rationale to use these MST metrics is that these metrics showed significant associations with cognitive decline and thalamic atrophy in previous empirical network studies in MS (Tewarie et al., 2014a, 2015a, 2014b).

### 2.3 Experiments

Given the clinical and cognitive impact of white matter, cortical, and thalamic degeneration, we focus on the effect of these types of damage on connectivity and networks. As the initial parameters of the model were obtained after parameter estimation based on empirical data from healthy controls (Robinson et al., 2001), we consider these parameter settings as our initial working point. For each experiment described below, we simulate network activity followed by computing the outcome measures. All simulations were repeated 50 times, and the mean and standard deviations across 50 realizations for each iteration were visualized in a graph. We follow a three‐step approach.

#### 2.3.1 White matter damage

WM damage was modeled by nonrandom and systematic reduction of the white matter connections between units up to 14% in known predilection sites (Daams et al., 2016; Li et al., 2013) (*n* = 24 and *n* = 202). A reduction of 14% was based on the percentage change in structural connectivity strength that can be observed when comparing empirical data of MS patients (*n* = 39) compared to those of healthy controls (*n* = 39) (see figure 3c in Shu et al., 2011).

#### 2.3.2 Cortical degeneration

Cortical degeneration was modeled by linearly decreasing the strength of within cortical unit connections. The reductions were informed by empirical values measured in a study on cortical thickness in longstanding MS that used exactly the same parcellation scheme as in the current modeling study (*n* = 102) (Tewarie et al., 2014a, 2014b). Data from this study were a subset of the data used in a recent paper on nonrandom patterns of cortical atrophy in MS (*n* = 208) (Steenwijk et al., 2015). The maximum reduction for every region was based on the relative decrease (%) in cortical thickness in MS patients compared to healthy controls (see Supporting Information, Figure S1 for regional cortical thickness values).

#### 2.3.3 Thalamic degeneration

Thalamic degeneration was modeled by linearly decreasing the strength of within thalamic unit connections. The reductions were informed by thalamic volumes measured in an empirical study in longstanding MS (*n* = 202) (Daams et al., 2016). The maximum reduction was equal to the relative reduction (%) in thalamic volumes in MS patients compared to healthy controls (100% − (18.50/20.78 × 100%) = 11%, see table 1 in a previous study) (Daams et al., 2016).

As within‐unit connections in both the cortex and the thalamus correspond to neuronal connection densities (Robinson et al., 2001), alteration of these parameters in experiments 2 and 3 correspond more to neuronal/axonal/synaptic loss (neurodegeneration/atrophy) within the cortex than demyelination, which is not modeled in this study. The inclusion of neuronal/axonal loss can be justified given findings from a recent study demonstrating that MRI‐measured atrophy in MS is driven by neuronal/axonal loss (Popescu et al., 2015). Modeling of grey matter demyelination would require within‐unit delays, which is computationally not straightforward and requires future studies. In addition, the stronger association between neurodegeneration and disability justifies focusing on that aspect of the disease in the current study rather than demyelination (Geurts & Barkhof 2008).

## 3 RESULTS

### 3.1 White matter damage

**Figure 2:**
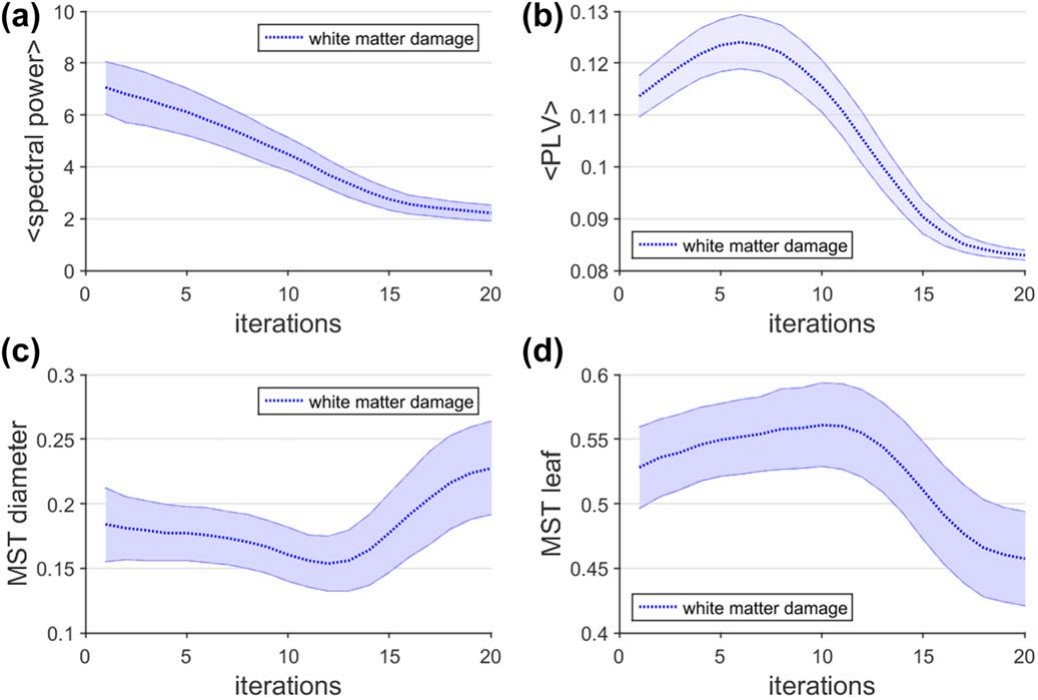
Effect of white matter damage on function. The effect of white matter damage on neuronal activity (a), functional connectivity (b), and functional network organization is shown (c,d). Dashed lines correspond to mean values across realizations for every iteration, and the shaded area around these dashed lines corresponds to the standard deviation across realizations, which can be interpreted as heterogeneity [Color figure can be viewed at http://wileyonlinelibrary.com]

The effect of white matter damage on functional activity, connectivity, and network organization is shown in Figure 2. Increasing white matter damage induces a monotonic decrease in mean neuronal activity, while functional connectivity changes are characterized by an inverted U‐curve, meaning that white matter damage initially causes an increase in connectivity, followed by a subsequent decrease. This inverted U‐curve is explained by damage to long range tracts that initially leads to higher local connectivity (i.e., connectivity of local groups is not perturbed by distant groups), followed by a global collapse of the network associated with lower connectivity. The effect of white matter damage on network integration and segregation remains small during the initial stages, but subsequently features a sudden and steep change during the transitional period between increased and decreased connectivity. The increased MST diameter (Figure 2c) can be interpreted as a decrease in network integration, whereas the decrease in MST leaf fraction (Figure 2d) can be interpreted as an increased network segregation. Figure 2c could give the impression that the initial stage of stable MST diameter values (during 0–10 iterations), is actually characterized by a decrease in MST diameter. However, note that there is still a large overlap between the standard deviations during this regime, and therefore this slight decrease cannot be considered as significant.

### 3.2 Grey matter damage: Cortical damage

**Figure 3:**
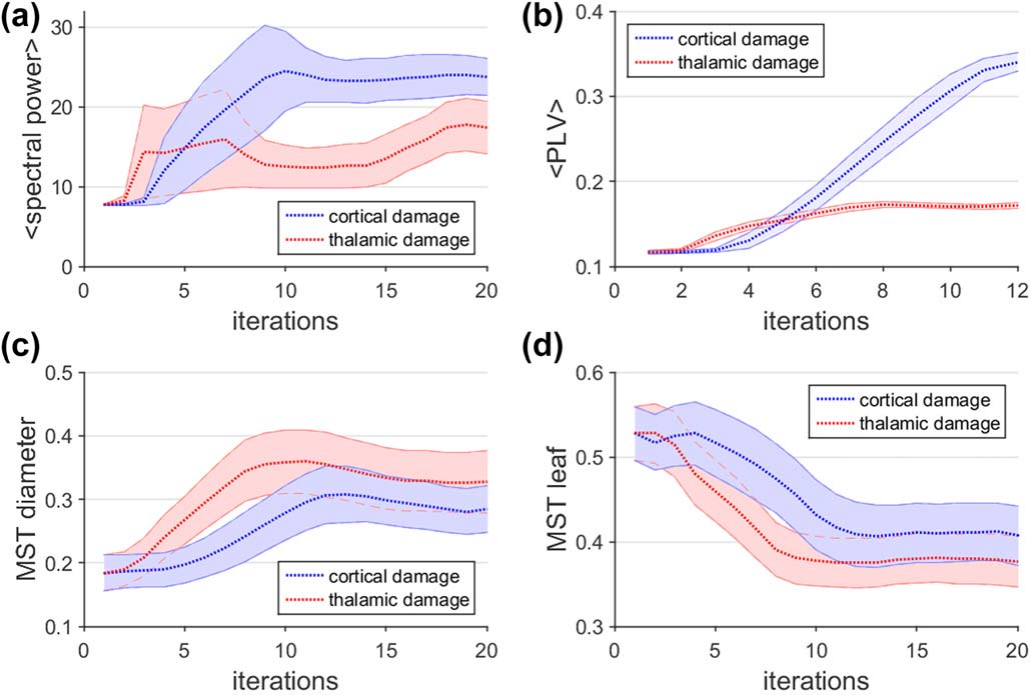
Effect of grey matter damage on function. The effect of grey matter damage on neuronal activity (a), functional connectivity (b), and functional network organization is shown (c,d). The blue curves correspond to the effect of cortical damage and the red curve to the effect of thalamic damage. Dashed lines correspond to mean values across realizations for every iteration, and the shaded area around these dashed lines corresponds to the standard deviation across realizations, which can be interpreted as heterogeneity [Color figure can be viewed at http://wileyonlinelibrary.com]

Simulations for the effect of cortical damage on functional activity, connectivity, and network organization are shown in Figure 3 (blue traces). Increasing cortical degeneration induces initially an increase in cortical neuronal activity (Figure 3a), followed by a plateau. Increasing cortical damage also brings about an increase in cortical functional connectivity (Figure 3b), but note that functional connectivity hardly changes in the initial phases of cortical damage. However, the effect of cortical matter damage on activity and connectivity is very distinct compared to the effect of white matter damage on these functional outcome measures. This discrepancy is however not very evident with respect to functional network organization. For cortical damage we can observe that MST diameter increases with increasing damage, that is, the networks become less integrated. The difference with white matter damage is, however, that this increase follows very quickly after the initial stages of damage, in contrast to the effect of white matter damage on the MST diameter, which initially stays quite stable. MST leaf fraction shows a rapid decrease after a short stable phase. Again the sign of the change is similar to that of white matter damage, being again the difference that network segregation is less robust against cortical damage than white matter damage.

### 3.3 Grey matter damage: Thalamic damage

Simulations for the effect of thalamic damage on functional activity, connectivity and networks are shown in Figure 3 (red traces). Similar to cortical damage, increasing thalamic degeneration induces an increase in neuronal activity, however, less pronounced than for cortical damage. Change in neuronal activity in the cortex during the course of thalamic damage follows a nonlinear effect with two maxima over time. Thalamic degeneration also brings about an increase in mean cortical functional connectivity, which occurs earlier during the course than for cortical damage, but thalamic damage has a less strong effect on cortical functional connectivity than cortical damage. However, thalamic damage seems to induce a comparable effect on network integration (MST diameter) and segregation (MST leaf fraction) to cortical damage, with the difference of a steeper slope for thalamic damage and a lack of an initial stable phase.

## 4 DISCUSSION

In this study, we used biophysical modeling to investigate the separate effects of white matter, cortical and thalamic degeneration on functional connectivity/activity and network organization in MS. We observed a nonlinear effect of white matter damage on functional connectivity, in the form of an initially increased, but subsequently decreased overall brain connectivity. Grey matter damage only resulted in increased connectivity, especially for simulated cortical degeneration. At the same time, both white and grey matter degeneration led to decreased network integration and segregation, albeit occurring at a faster pace for grey matter degeneration. Taken together, these findings could explain the heterogeneity of connectivity results in the literature.

Our simulations demonstrated that white matter damage can induce both an increase and decrease in functional connectivity depending on the amount of white matter damage (in terms of axonal/neuronal damage). This inverted U‐curve curve of functional connectivity during the course of increasing white matter damage may explain its inconsistent relationships with clinical outcome measures (Rocca et al., 2015b, 2010; Sbardella et al., 2016; Tona et al., 2014). This would especially be the case for cross‐sectional studies, where patients with different amounts of white matter damage could be found on different points along the curve in Figure 2b. At the same time, functional network organization seems to be fairly robust against white matter damage up to a critical point after which the functional network undergoes a rapid disintegration.

In contrast to the white matter, damage of the gray matter induced functional network disintegration at an earlier stage, that is, less cortical damage is required to lead to disintegration of functional networks. Apparently, functional network organization is less robust for grey matter damage than for white matter damage. This could explain why cognitive and clinical measures correlate more strongly to grey matter than white matter alterations in MS. The discrepancy between damage *within* the nodes (i.e., atrophy) and damage *between* the nodes (i.e., structural connections) on network organization seems to be a generic principle in a diverse range of networks, which is related to the concept of degeneracy (Fornito, Zalesky, & Breakspear, 2015): due to the fact that the brain has many more structural connections than nodes (e.g., nodes are usually connected to several other nodes), network function can be retained in the presence of damage *between* the nodes by using one of the many alternative connection routes. The pool of alternatives for damage *within* the nodes (i.e., in the gray matter) is much smaller (i.e., there is less redundancy for grey matter than for white matter connections), which could explain its sensitivity for network disruption.

Our findings have important implications. First, our results demonstrate that an increase in connectivity and activity can occur as a result of structural damage and thus not solely as a result of a compensatory process. Therefore, caution should be exercised when interpreting increased functional connectivity values in cross‐sectional studies as “compensation” (in the sense that it is a beneficial, teleological response to damage). Second, as demonstrated by the fast transitions in Figures 2c,d and 3c,d, depending on the type and amount of damage, changes in functional network integration and segregation measures are disproportionate to the amount of damage. Little damage may cause large changes in network topology. Third, given the observed specific effects of different types of damage, functional connectivity changes in the beginning of the disease might be different compared to later stages of the disease, as it is known from empirical data that thalamic atrophy is present in the earliest phases of the disease (Minagar et al., 2013), while cortical atrophy is thought to be more prominent and developing in patterns in later stages (Fisniku et al., 2008). Whether the supposed sequential occurrence of thalamic and cortical atrophy is driven by one of the changes remains to be investigated. In contrast to functional connectivity, the direction in which network integration and segregation changes, seems to be unambiguous for white and grey matter damage, indicating that it may be more fruitful to analyze network topology measures in empirical studies as a correlate for cognitive and clinical outcomes.

Some methodological aspects of our study warrant discussion. First, our results apply to resting‐state functional networks and not task‐based connectivity findings, which requires a different way of modeling in terms of external inputs that drive the dynamics. Second, all large‐scale computational models lack regional specificity along white matter tracts. Thus we cannot distinguish between lesional damage and diffuse white matter damage along a white matter tract. Finally, we did not investigate the effects of heterogeneity of conduction velocities along white matter tracts to simulate diffuse white matter demyelination, and did not use thalamo‐cortical connectivity as an outcome measure, warranting future studies. Last, the empirical data of the different types of structural damage that has been used to inform the simulations differs in disease duration. This will have influenced the end points (last iteration) in the graphs (Figures 2 and 3), but not the shape of the curves.

In conclusion, we have demonstrated that joint white and grey matter damage can induce concurrent increases and decreases in functional connectivity, warranting caution when interpreting such changes as a compensatory mechanism. In addition, these concurrent increases and decreases in connectivity could also explain the reported heterogeneity of functional connectivity results reported in the literature. Functional network topology measures show a more insightful pattern of change, regardless of the type of damage induced, which enforces their value for future imaging studies in MS.

## Supporting information

Additional Supporting Information may be found online in the supporting information tab for this article.

Supporting Information
