# Supplementary material for: Explaining the heterogeneity of functional connectivity findings in multiple sclerosis: An empirically informed modeling study
Source: Hum Brain Mapp. 2018 Feb 21;39(6):2541–8. doi: 10.1002/hbm.24020 (PMC5969233; doi:10.1002/hbm.24020)
Supplement: Supplementary file 1 — Supporting Information [file HBM-39-2541-s001.docx]

**Supplementary information**

***Table S1:*** *model parameters for cortico-thalamic mean field model. Parameters are the same as in* [*^8-10^*](#_ENREF_8)*, except that the inhibition to excitatory populations was slightly higher tuned to balance the effect of the excess of external excitatory activity coming from the network.*

| *Model parameters* | *values* |
| --- | --- |
| *Q_max_* | *250 s^-1^* |
| *θ* | *15 mV* |
| *σ* | *3.3 mV* |
| *α* | *50 s^-1^* |
| *β* | *200 s^-1^* |
| *γ* | *100 s^-1^* |
| *ν_ee_* | *1.2 mV s* |
| *ν_ei_* | *-1.9 mV s* |
| *ν_es_* | *1.2 mV s* |
| *ν_ii_* | *-1.8 mV s* |
| *ν_ie_* | *1.2 mV s* |
| *ν_is_* | *1.2 mV s* |
| *ν_sr_* | *-0.8 mV s* |
| *ν_se_* | *1.2 mV s* |
| *ν_re_* | *0.4 mV s* |
| *ν_rs_* | *0.2 mV s* |
| *τ_ct_* | *0.04 s* |

***Figure S1:*** *The upper panel shows mean cortical thickness for every region in the automated anatomical labelling atlas (AAL) for 102 longstanding MS patients used in a previous study. Subject characteristics and demographics can be found in this previous paper.*[*^7^*](#_ENREF_7) *Cortical thickness for all 78 cortical regions for healthy controls is shown in the middle panel, whereas the relative differences between the mean cortical thickness values between healthy controls and patients is shown in the bottom panel.
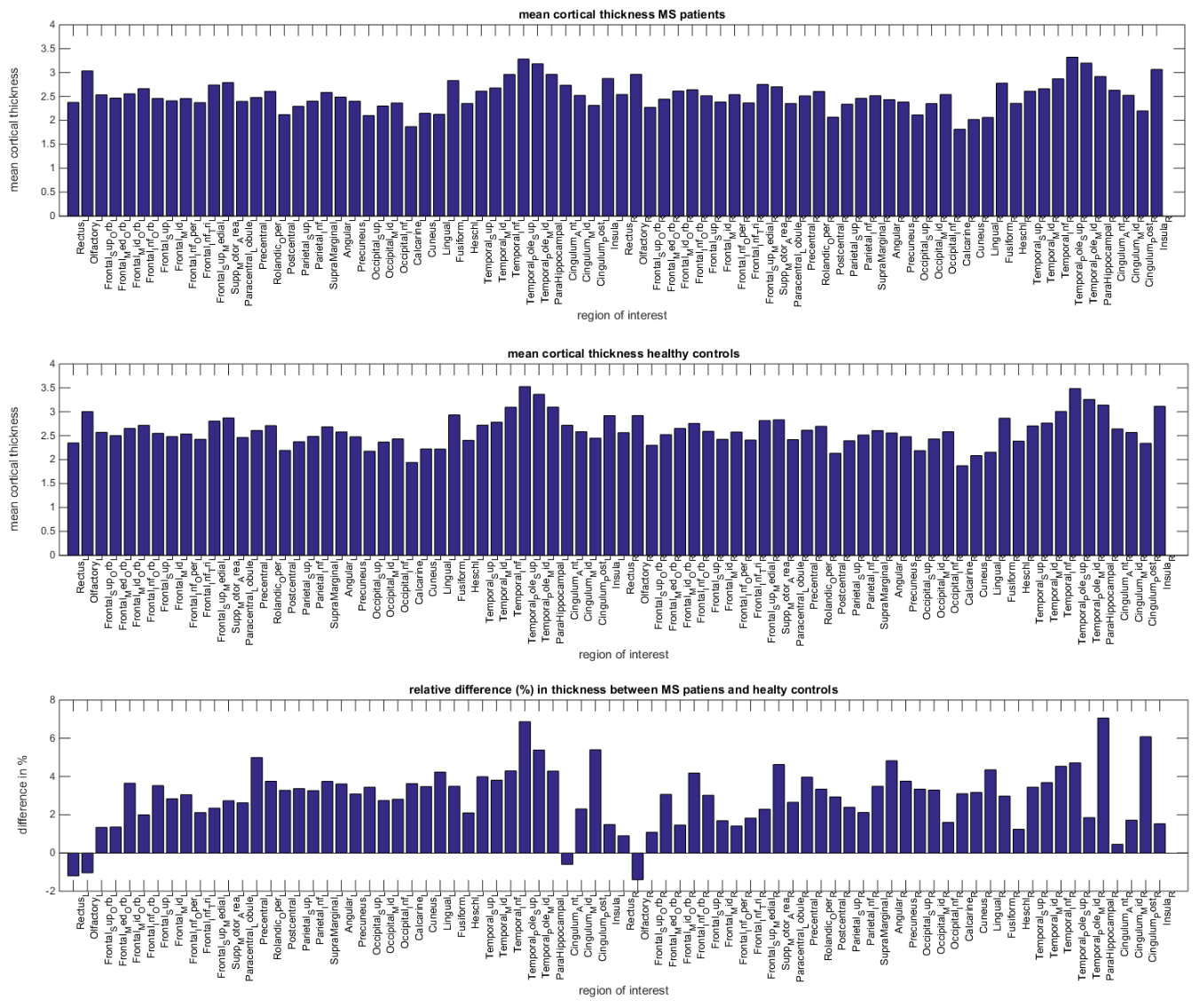
*
